# Mechanisms of Cardiovascular System Injury Induced by COVID-19 in Elderly Patients With Cardiovascular History

**DOI:** 10.3389/fcvm.2022.859505

**Published:** 2022-05-04

**Authors:** Yaliu Yang, Mengwen Yan

**Affiliations:** Department of Cardiology, China-Japan Friendship Hospital, Beijing, China

**Keywords:** COVID-19, cardiovascular system injuries, renin-angiotensin system (RAS), inflammation, immune dysregulation, endothelial injury

## Abstract

The coronavirus disease-2019 (COVID-19) pandemic, caused by severe acute respiratory syndrome coronavirus (SARS-CoV-2), represents a great threat to healthcare and socioeconomics worldwide. In addition to respiratory manifestations, COVID-19 promotes cardiac injuries, particularly in elderly patients with cardiovascular history, leading to a higher risk of progression to critical conditions. The SARS-CoV-2 infection is initiated as virus binding to angiotensin-converting enzyme 2 (ACE2), which is highly expressed in the heart, resulting in direct infection and dysregulation of the renin-angiotensin system (RAS). Meanwhile, immune response and hyper-inflammation, as well as endothelial dysfunction and thrombosis implicate in COVID-19 infection. Herein, we provide an overview of the proposed mechanisms of cardiovascular injuries in COVID-19, particularly in elderly patients with pre-existing cardiovascular diseases, aiming to set appropriate management and improve their clinical outcomes.

## Introduction

In December 2019, pneumonia of unknown cause was reported in Wuhan, China (1). By early January 2020, sequencing analysis indicated the pathogen as a novel coronavirus, severe acute respiratory syndrome coronavirus 2 (SARS-CoV-2), and the related clinical syndrome was named coronavirus disease-2019 (COVID-19) (2). It had spread rapidly throughout the world and on 11 March, 2020, the World Health Organization (WHO) declared COVID-19 a global pandemic. This infection has brought a great threat to healthcare and socioeconomics worldwide.

Whereas COVID-19 is characterized by respiratory symptoms, Huang et al. reported that 12% of patients present acute cardiac injury, defined as an ejection fraction decline and troponin I elevation (3), with a wide spectrum of clinical manifestations ranging from an acute coronary syndrome, myocarditis, arrhythmia, to cardiac dysfunction. Accumulating evidence reveals that acute cardiovascular injury is associated with increased severity and mortality of COVID-19 (4). Recent literature on long-term sequelae of COVID-19 shows prolonged cardiovascular damage in a large proportion of post-COVID-19 patients (5), for example, parasympathetic overtone and increased heart rate variability (6).

People with underlying cardiovascular diseases are prone to develop severe conditions, even in pediatric patients. The COVID-19-infected children with congenital heart disease represent worse clinical courses when compared to healthy control (7). Likewise, the elderly people with pre-existing cardiovascular comorbidities, who are more susceptible to cardiac injuries of COVID-19, are at a higher risk of poorer prognosis. In this review, we emphasize the pathogenesis of COVID-19-induced cardiovascular injury, particularly in elderly patients with underlying cardiovascular diseases.

## The Angiotensin-Converting Enzyme 2 Receptor and Renin-Angiotensin System

As with SARS-CoV, ACE2 has been established as the dominant route of entry for SAR-CoV-2 upon binding of the viral spike protein (S protein) (8). In the process, furin, a proprotein convertase, cleaves S protein into two activated subunits, namely, S1 and S2. The S1 domain binds the ACE2 receptor through its receptor-binding domain (RBD), while the S2 subunit is necessary for virus-cell fusion after further processed by transmembrane serine protease 2 (TMPRSS2) (9). Compared to SARS-CoV, SARS-CoV-2 exhibits potent binding to ACE2 and immune evasion because of greater affinity of RBD, protease pre-activation of the spike, and hidden RBD, all of which in turn results in higher transmissibility of SARS-CoV-2 (9, 10). Until now, SARS-CoV-2 has undergone substantial evolution, for instance, SARS-CoV-2 Delta variant, one of the predominant circulating strains, exhibits higher infectivity, on a basis of increased ACE2 interaction owing to more RBD-up states and Delta T478K substitution (11). The virus-ACE2 binding actuates the virus-cell fusion, virus replication, and ACE2 loss at the same time.

Angiotensin-converting enzyme 2, a type I integral membrane protein, acts as a transmembrane protein or a soluble catalytic ectodomain *in vivo* (12). The transmembrane ACE2 can be measured as ACE2 expression, and studies indicate that the transmembrane ACE2 is abundant in lungs, heart, and endothelial cells (ECs) (13, 14). Chen et al. delineate ACE2 expression in cardiac resident cells, particularly in pericytes, a type of perivascular mural cells. Pericytes support capillary EC function and are associated with myocardial microcirculation (15). Pathology analysis of COVID-19 infections reveals direct viral infection and diffuses inflammation of the ECs, which may attribute to coronary plaque disruption and thrombosis (16). In this regard, direct viral infection of cardiac tissue implicates the cardiac complications of COVID-19 infection. A disintegrin and metalloproteinase domain-containing protein 17 (ADAM17) mediates cleavage and shedding of the soluble ACE2 ectodomain. ACE2 ectodomain, also known as soluble ACE2, can be detected as plasma ACE2 activity (12). The soluble ACE2 has been recently recognized to help in controlling SARS-CoV-2 infection *via* inhibiting their interaction with cell-bound ACE2 (17). The circulating ACE2 has been shown to correlate with cardiac remodeling, endothelial dysfunction, and is a predictor of major adverse cardiovascular events (18).

Beyond the host receptor in SAR-CoV-2 infection, ACE2 is an important component of the RAS. The circulating RAS system is finely controlled by complex feedback to maintain blood volume. Commonly, the action of Angiotensin (Ang) II on Ang II type 1 receptor (AT_1_R) stimulates aldosterone secretion regulated by renin in response to homeostatic demand. On the other hand, tissue angiotensin system has been identified since prorenin expression was found in many organs, tissues, such as heart, lungs, and brain. Thereafter, other biological effects of RAS have been recognized. Prorenin, firstly activated through proteolytic enzymes or unfolded by binding with (pro)renin receptor, converts the substrate angiotensinogen (AGT) to Ang I (10, 17). Ang I is cleaved to Ang II, the most active agent in RAS by ACE or chymase, the major catalytic enzyme in the heart (19). Ang II acts on two G-protein coupled receptors, AT_1_R and type 2 receptor (AT_2_R). Ang II/AT_1_R binding exerts vasoconstriction, pro-inflammatory, pro-fibrotic, and proliferative effects through various intracellular protein signaling pathways, such as tyrosine kinases, serine/threonine kinases, mitogen-activated protein kinase (MAPK) family, and various protein kinase C isoforms, while Ang II/AT_2_R interaction, with a much less affinity when compared to Ang II/AT_1_R action, activates various protein phosphatases, the nitric oxide (NO)/cyclic GMP system, and phospholipase A_2_, counteracting AT_1_R actions (20–22). ACE2 mediates Ang (1–7) generation from Ang II. Ang (1–7) acts *via* AT_2_R, Mas receptor (MasR), and Mas-related G protein-coupled receptor D (MgrD) and performs protective actions of anti-inflammation, vasodilation, and anti-fibrotic effects (10, 23). As such, Ang II degradation and Ang 1–7 generation accelerate cardiovascular protection. Previous animal studies showed that ACE2 inactivation was correlated with reduced cardiac contractility, coronary vasoconstriction, microvascular dysfunction, and less myocardial blood flow (24, 25).

**Figure 1:**
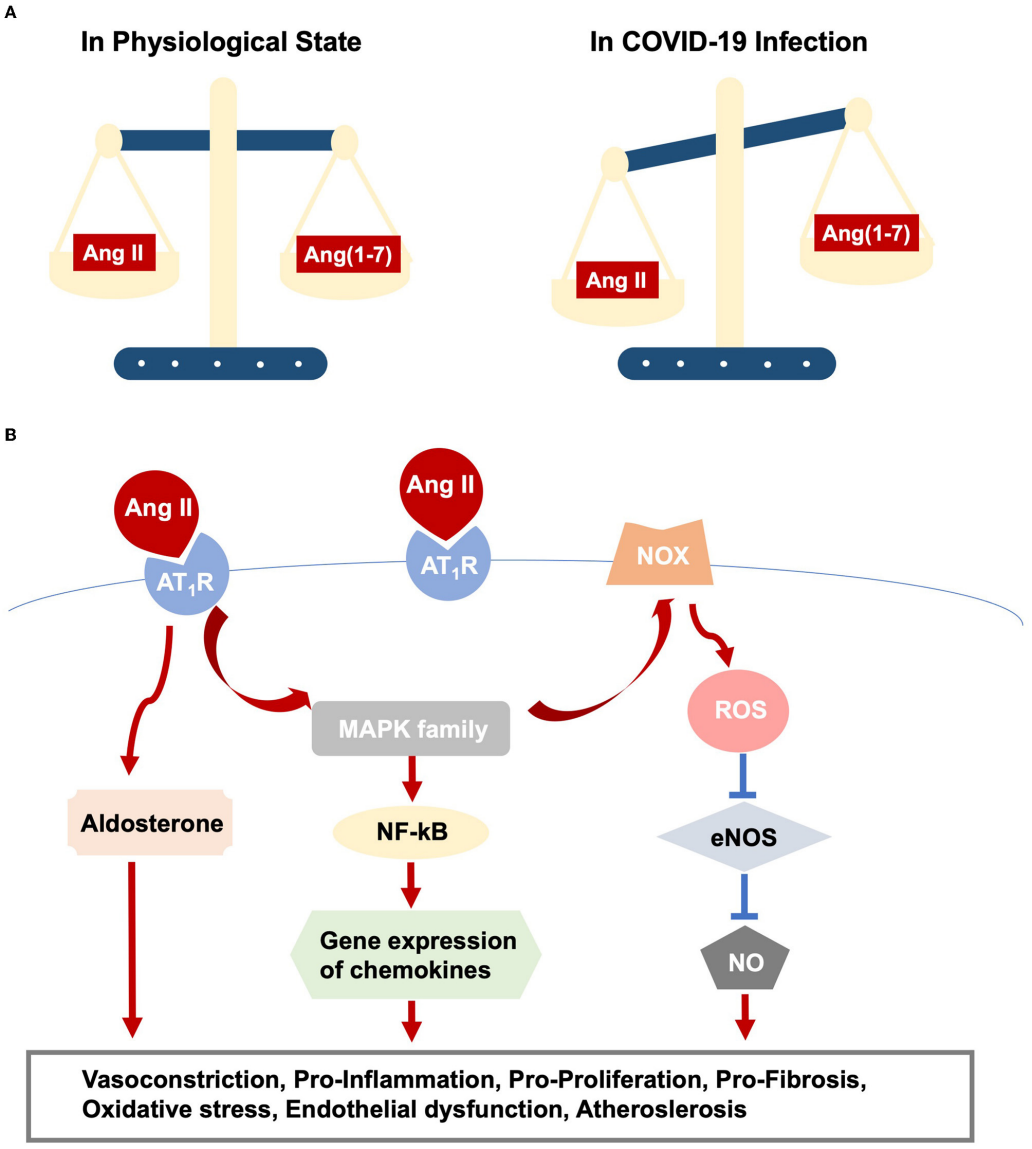
RAS dysfunction in COVID-19 infection. **(A)** Outlines imbalance between Ang II/AT_1_R and Ang(1-7)/Mas action. **(B)** Elucidates intracellular pathways upon Ang II/AT_1_R interaction, which ultimately result in various cardiac and vascular injuries.

Virus-ACE2 internalization in COVID-19 infection leads to ACE2 destruction. The ACE2 deficiency modulates the imbalance of Ang II/Ang (1–7) and thus amplifies Ang II/AT_1_R actions (Figure 1A). Moreover, aging-related RAS alteration contributes to cardiac dysfunction in COVID-19 infection. A previous study discovers increased cardiac Ang II formation in a chymase-driven manner in aged rats (26). Ang II level *per se* mediates ACE and ACE2 expression, with higher ACE and lower ACE2 production, which in turn leads to outweighed Ang II/AT_1_R interaction (27). In addition, animal studies display upregulation of AT_1_R in both the aging heart and vasculature. On the other hand, AT_2_R was highly expressed in fetal tissues but dropped to a comparatively low level in adulthood. The altered ratio of AT_1_R and AT_2_R might increase blood pressure and induce inflammation (22). Elderly patients with pre-existing cardiovascular diseases reported the increased ACE2 expression, promoting vulnerabilities to COVID-19 and direct viral damage. Noteworthy, RAS inhibitors, frequently medicated to older patients with cardiovascular diseases, are safe, despite increased membrane-bound ACE2 expression (28).

## Immunity and Inflammation

After initial infection with the virus, the innate immune signaling activates as the first-line defense, mediating virus recognition, killing virus-infected cells, stimulating inflammation, and adaptive immunity. The adaptive immune system, consisting of T and B cells, neutralizes viral particles, clears the virus, and sets long-term immunity.

Upon COVID-19 infection, pattern recognition receptors (PRRs) of antigen-presenting cells (APCs) detect pathogen-associated molecular patterns (PAMPs), namely, viral RNA and spike proteins as the main PAMPs in the case of SARS-CoV-2. Interaction of PAMPs with PRRs, such as membrane-bound Toll-like Receptors (TLRs), or cytosolic RIG-I-Like Receptors (RLRs), alongside the recruitment of cytoplasmic molecular adapters, such as MyD88, stimulates a variety of signaling cascades, mediating cytoplasmic transcription factors, such as nuclear factor kappa B (NF-KB), interferon regulatory transcription factor 3 (IRF3) translocating toward nuclear (29, 30). NF-KB facilitates the expression of genes in innate and adaptive immune response, as well as the development of cytokine storm (31, 32).

Subsequently, the immune cells produce cytokines, such as interferons (IFNs), interleukins (ILs), chemokine, and tumor necrosis factor (TNF), exerting broad antiviral effects (33). Type 1 IFNs, produced at the early stage of SARS-CoV-2 infection, exhibit pivotal antiviral effects by promoting apoptosis of virus-infected cells and antigen presentation to T cells *via* the induced expression of major histocompatibility complex class I (MHC I) (34, 35). However, SARS-CoV-2 produces multiple interferon antagonists and impairs IFN actions, resulting in viral replication, inflammation, and hypercytokinemia, which are considered the main causes of COVID-19 severity (32).

The adaptive immune response also plays a pivotal role in virus defense. B cells release virus-specific antibodies with the help of CD4+ T cells while CD8+ T cells mediate direct apoptosis of virus-infected cells (36). In the process, antigen presentation by APCs is essential for the adaptive immune response of T and B lymphocytes. However, COVID-19 infection is characterized by lymphopenia (37). Probably, SARS-CoV-2 exerts immune evasion through impaired maturation of dendritic cells, leading to hampered dendritic cells (DCs) homing to lymph nodes and failure of T lymphocytes activation (38).

In addition to direct viral infection, exacerbated inflammatory drivers and dysregulated cell-mediated response contribute to cardiovascular injuries in COVID-19 infection. Noteworthy, the elderly population are vulnerable to cardiovascular injuries and poor prognosis, partly attributed to the age-related changes of the immune system. Aging is well-characterized by chronic inflammatory responses in the absence of infection, also called inflammaging (39). Proper inflammation is necessary for pathogen clearance and tissue repair, whereas inflammaging is associated with tissue damage and disease. Meanwhile, with age, there is a decline in both the count and functionalities of immune cells. The DCs from aged mice and humans are less efficient to migrate, secret cytokines, and prime T cells in viral defense (40, 41). Less production of new lymphocytes was observed in the aged population (42). Sex hormones participate in immune activities directly, through the expression of estrogen or testosterone receptors on immune cells, such as lymphocytes and macrophages (17). The hormonal changes with aging may, to some extent, elucidate the age-related changes of the immune response.

## Endotheliopathy and Coagulopathy

Endothelial dysfunction and coagulopathy are hallmarks of COVID-19 infection. The increased levels of von Willebrand factor (VWF) antigen, D-dimers, and tissue plasminogen activator are reported in the COVID-19 group, substantiating endothelial damage and pro coagulation in COVID-19 infection (43, 44). Autopsy cases identify lymphocytic endotheliitis, frequent microthrombi as well as venous and arterial thromboembolism (16, 45, 46). A recent study indicates persistent endothelial damage in post-COVID-19 patients; on the other hand, Charfeddine et al. demonstrate that the lasting endothelial dysfunction is an independent risk factor of long COVID-19 syndrome (47, 48).

The wide distribution of ACE2 on ECs makes it a direct target for SARS-CoV-2 entry (49). Virus-cell binding downregulates membrane ACE2, resulting in reduced degradation of Ang II and decreased production of Ang (1–7). Subsequently, the mounting Ang II/AT_1_R interaction exhibits pro-inflammatory cytokines secretion and pro-thrombotic actions by limiting NO and prostacyclin release. Otherwise, ACE2 regulates the kinin-kallikrein systems and participates in the inactivation of circulating bradykinin (BK). In this regard, ACE2 loss in COVID-19 infection leads to an increased level of BK, which induces EC activation and dysfunction, together with increased vascular permeability (50, 51). Exposure to SARS-CoV-2 spike protein *in vitro* stimulates caspase and apoptosis in ECs, whereby the loss of endothelial integrity triggers hypercoagulation (51).

The vascular endothelium participates in immune response and inflammation. Cytokines, such as IL-6, activate ECs, and in turn, the activated ECs express plenty of adhesion molecules, i.e., intercellular adhesion molecule-1 (ICAM-1) and vascular cell adhesion molecule-1 (VCAM-1), resulting in the recruitment of leukocytes and platelets. In addition, ECs express different TLRs, mediating PAMPs recognition and antigen presenting to T cells (52). Regulated EC activation helps in limiting pathogen invasion, whereas the hyperinflammatory profile, often seen in the severe COVID-19 cases, promotes profound endothelial dysfunction and damage, contributing to multiple organ failure (46).

Resting ECs also participate in the dynamic interplay between coagulation and fibrinolysis. Direct SAR-CoV-2 infection induces endothelium injury and apoptosis, decreasing its antithrombotic activity. In addition, in the setting of inflammation, inflammatory molecules or injured ECs stimulate coagulation by increasing tissue factor (TF) expression by monocytes and ECs *in vitro* (53). TF and its downstream activated factors ultimately stimulate the coagulation cascade and produce clots (54). In addition, SARS-CoV-2 infection, together with SARS and Middle East respiratory syndrome (MERS), is correlated with thrombocytopenia (55). One explanation is that platelets are hyper-activated in these viral-infected patients, probably owing to hypoxia, immune responses, and endothelial dysfunction in the case of COVID-19 (16, 55). The activated platelets interact with leukocytes, contributing to the leukocyte cytokine release, such as CC-chemokine ligand 2 (CCL2), CCL3, IL-1β, and the release of neutrophil extracellular traps (NETs) wrapped with TFs, which in turn activates the extrinsic coagulation cascade resulting in thrombin formation (56, 57). Terminal complement components, such as the C5b-9 (membrane attack complex) and the C4d, have been discovered in the microvasculature, suggesting the association of complement system with microvascular injury in COIVID-19 (58).

Indeed, cardiovascular complications of COVID-19 are highly prevalent and contain acute cardiac injury, myocarditis, and a hypercoagulable state, all of which may be influenced by endotheliopathy and coagulopathy. Age is the main risk factor for COVID-19-related death and intensive care unit (ICU) admission. Age-associated EC dysfunction might be the reason for the poor prognosis in the elderly, leading to vascular pathologies and cardiovascular diseases. Abundant evidence demonstrates that the impaired endothelium-dependent NO-mediated vasodilation is associated with cardiovascular events, and the findings that endothelial nitric oxide synthase (eNOS)-deficient mice display a premature cardiac aging phenotype together with early mortality indicate the critical role of endothelium-derived NO on cardiovascular protection in aging (59, 60). The reduced bioavailability of NO contributes to age-associated impairment of angiogenesis, leading to ischemic tissue injury, such as myocardial ischemia and infarction (61). Csiszar et al. (62) show with advancing age, coronary arteries undergo pro-inflammatory alterations, age-related decline in NO bioavailability as well as upregulation of TNFα and caspase 9, promoting endothelial apoptosis.

## COVID-19-Related Cardiovascular Complications in Elderly

**Figure 2:**
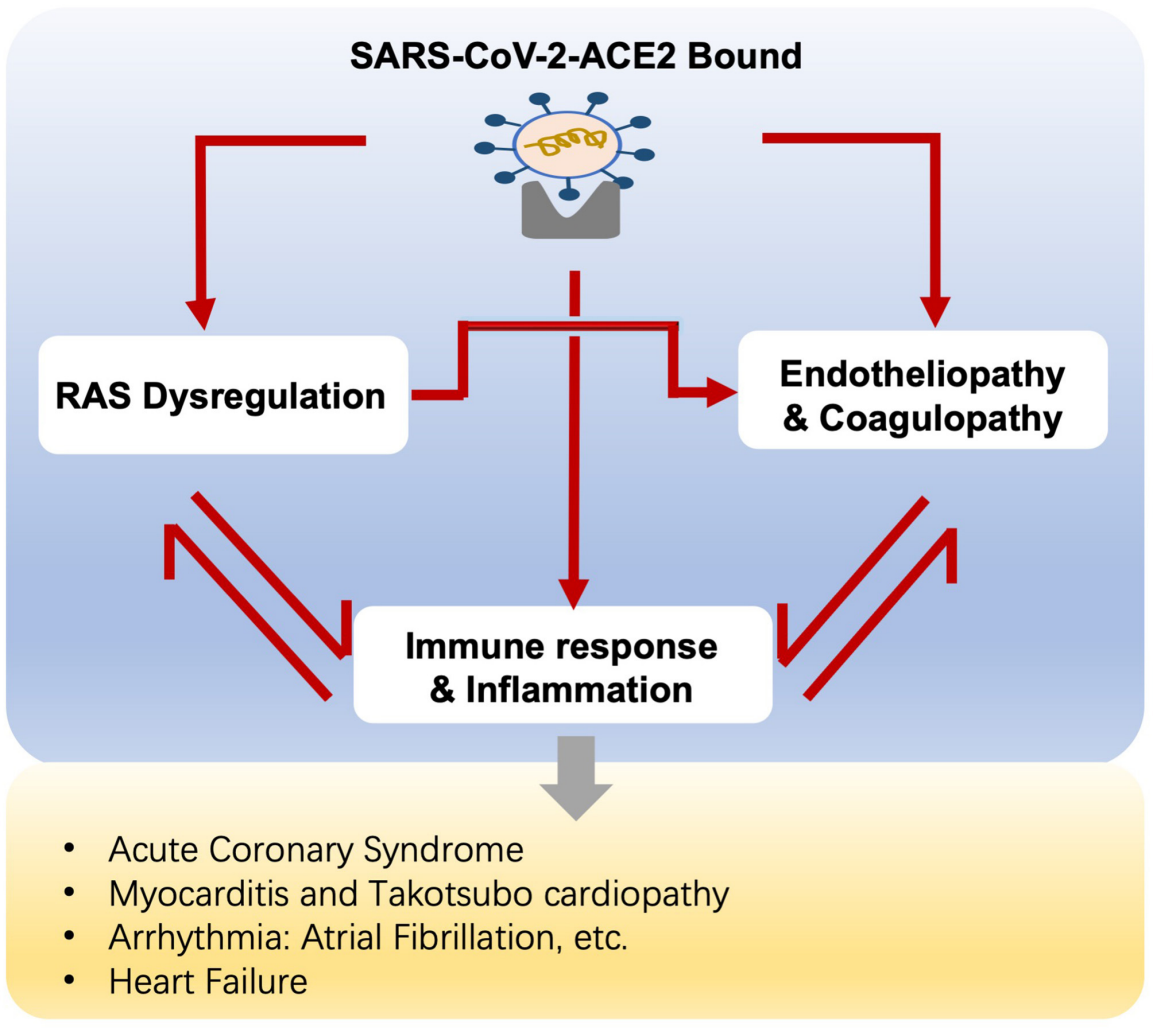
Potential mechanisms of cardiovascular complications in COVID-19 patients.

A variety of cardiovascular complications are documented in COVID-19, ranging from myocardial injury, myocarditis, arrhythmia, to cardiac dysfunction and heart failure. The crosstalk between RAS, hyper-inflammation, endotheliopathy, and coagulopathy accounts for the mechanism of cardiovascular involvement in COVID-19 (Figure 2).

- Direct viral infection induces myocardial injury, whereas virus-ACE2 binding brings overactive Ang II/AT1R actions, resulting in vasoconstriction and increased blood pressure because of its role as an endocrine regulator. Additionally, activated Ang II/AT_1_R interacts with multiple intracellular signaling, for example, the MAPK family (63), and regulates the inflammatory process. In this sense, AT_1_R triggers NF-kB activation, which promotes the gene expression of chemokines, cytokines, and adhesion molecules (64). The immune response and hyper-inflammation concerning SARS-CoV-2 infection, partly owing to Ang II/AT_1_R action, lead to cardiac and vascular remodeling, as well as atherosclerotic plaque growth and rupture (63, 65) (Figure 1B).
  - Likewise, Ang II promotes oxidative stress and endothelial dysfunction *via* action on AT_1_R and the downstream phagocytic nicotinamide adenine dinucleotide phosphate (NADPH) oxidase (NOX) and reactive oxygen species (ROS) signaling (66), promoting lipid oxidation, macrophage uptake of lipids, and monocyte recruitment, leading to vascular inflammation and atherosclerosis (43, 67) (Figure 1B).
- In turn, previous studies show that the recruitment of immune cells, i.e., monocytes and macrophages, into the vascular wall strengthens the Ang II-induced endothelial dysfunction and inflammation (68, 69). At the same time, ECs participate in the SARS-CoV-2-induced immune response and hyper-inflammation, which in turn trigger endothelial injury as we discussed above.
- In addition, vascular smooth muscle cells (VSMCs), responsible for vascular homeostasis, also play a key role in disease progressions, such as hypertension and atherosclerosis. In the process, phenotypic switching of VSMCs has been considered of fundamental importance, transforming the contractile VSMCs to synthetic phenotypes, i.e., macrophage-like genotypes. Activated EC-VSMC interaction *via* inflammatory cytokines promotes the transition of VSMCs to macrophage-like phenotypes. Macrophage-like VSMCs acquire inefficient phagocytic functions and express different scavenger receptors, for example, low-density lipoprotein receptor-related protein 1, facilitating the influx of low-density lipoprotein, thus attributing to the formation of VSMC-derived foam cells and subsequent atherosclerotic plaque growth (70). Meanwhile, Ang II/AT1R action mediates proliferation and migration of VSMCs through phosphatidylinositol 3-kinase (PI3K)/Akt and MAPKs, affecting atherogenesis (71, 72). Therefore, we are assuming that VSMCs modulate COVID-19 progression and the relevant cardiovascular complications. To support that, more investigations are needed.

Aging is associated with dysregulated RAS, inflammaging, and endothelial dysfunction as we described above. Therefore, we speculate that RAS activation, immune and hyper-inflammatory actions, endotheliopathy, and coagulopathy, all of which mutually reinforce each other, together with the pre-existing aging-related dysregulations, unfold the underlying mechanisms of COVID-19 infection, and the contaminant cardiovascular complications in the elderly.

The incidence of COVID-19-related stroke, one of the most important primary cardiovascular outcomes, ranges from 1 to 6% in hospitalized patients with COVID-19 (73). Concerningly, stroke in patients with COVID-19 is associated with a poorer prognosis when compared to COVID-19 negative stroke patients (74). Moreover, COVID-19-related stroke is more often in the elderly population, particularly those with pre-existing disorders, such as hypertension, atherosclerosis, and atrial fibrillation (75). The pathogenesis of ischemic stroke, the dominant subtype of strokes, is multifactorial and similar to other arterial thromboses, it is developed in COVID-19. In this regard, the interplay of inflammation, coagulopathy, endotheliopathy, and platelet activation, together with cardioembolism, contribute to COVID-19-related ischemic stroke (73).

## Comorbidities

The elderly people often have to deal with various comorbidities, i.e., diabetes mellitus (DM), chronic kidney disease (CKD), dyslipidemia, all of which are risk factors of cardiovascular disease. In the case of COVID-19 infection, the interplay between the viral infection and the concomitant comorbidities might exacerbate COVID-19 outcomes, such as cardiovascular injuries.

Diabetes mellitus increases the risk of hospitalization, mortality, and need for critical care in COVID-19. The DM group with pre-existing systemic endothelial and microvascular dysfunction undergoes extra endothelial and microvascular impairment in COVID-19 infection and the “double-killing” results in worse prognosis and multiple organ failure (76).

The incidence of CKD increases with age, and 38% of the patients with CKD are more than 65 years old (77). Cardiovascular causes are recognized as the leading cause of death, accounting for 50%of the mortality in the CKD population (78). Therefore, it is of great importance to investigate the cardiovascular injuries in the elderly with CKD induced by the pandemic COVID-19 infection. A comprehensive review reveals the effect of CKD on increased hospitalization and mortality of COVID-19, perhaps owing to immune dysfunction and increased susceptibility to infections (77, 79).

Accumulating studies demonstrate that lipid disorders are associated with an increased risk of COVID-19 progression by 39% (80, 81). Although Petrilli et al. (82) show no correlation between dyslipidemia and prognosis of COVID-19. Cholesterol is an essential factor in lipid rafts, which are involved in the entry of SARS-CoV-2. Therefore, increased cholesterol level increases susceptibility to SARS-CoV-2 (83). On the other hand, COVID-19 alters lipid metabolism, characterized by a decrease in total cholesterol, high-density lipoprotein, low-density lipoprotein, and an increase in triglycerides (83).

## Conclusion

The COVID-19 pandemic has swept the world and brought significant loss of health, life, and livelihoods, especially in the aged and those with underlying cardiovascular diseases. To our current knowledge, the COVID-19 is initiated as the viral-ACE2, the dominant host receptor interaction, and the subsequent effects on RAAS signaling, immune system, endothelium, and thrombosis confer to the complex pathologies in the viral infection. The findings of ACE/ACE2 imbalance, dysregulation of immune responses, endothelial dysfunction, and angiogenesis impairment in the elderly might explain the more severe conditions and cardiovascular involved in the old patients of COVID-19 infection.

Consequently, during the course of treatment for COVID-19, medical experts/clinicians must pay particular attention to protecting the cardiovascular system. Elderly patients with cardiovascular disease will be encountered significant healthcare disparities that exist in their management, when compared with younger counterparts. While making therapeutic decisions, age should not be considered in isolation but rather as one of many factors in the comprehensive assessment model, keeping in mind patients' overall health, frailty, cognition, quality of life, estimated life expectancy, and above all preferences. We should pay close attention to the comorbidities, balance the risk of ischemia and bleeding, and carefully adjust the medication dose. Overall, elderly patients with a history of cardiovascular disease remain undertreated with evidence-based therapies, experience worse outcomes, and represent an opportunity for enhancing and mitigating healthcare disparities. Scientists have developed vaccines for the coronavirus, which bring promise to tackle the global pandemic of COVID-19, especially for elderly patients. In addition, close monitoring of cardiac function in elderly patients with COVID-19 can prevent, or at least limit, myocardial injury, thereby reducing mortality. Further studies are urgently needed to more clearly elucidate the pathophysiology, host/pathogen interactions, the host immune response, and heart phenotype characteristics of COVID-19-infected elderly patients. The underlying mechanisms of myocardial injury, diagnosis, related effective medical treatment strategies, and follow-up are required to advance targeted treatments and improve patient prognosis.

## Author Contributions

YY searched and selected the references, and wrote the first draft of the review. YY and MY contributed towards literature review and interpretation of the manuscript. MY helped to determine the content and structure of the review, and contributed to the writing and revision of the manuscript. All authors approved the final version of the paper.

## Funding

This study was funded by grants from the National Natural Science Foundation of China (81700262) and the Academic Project of China-Japan Friendship Hospital (Number 2019-2-QN-85).

## Conflict of Interest

The authors declare that the research was conducted in the absence of any commercial or financial relationships that could be construed as a potential conflict of interest.

## Publisher's Note

All claims expressed in this article are solely those of the authors and do not necessarily represent those of their affiliated organizations, or those of the publisher, the editors and the reviewers. Any product that may be evaluated in this article, or claim that may be made by its manufacturer, is not guaranteed or endorsed by the publisher.
